# The risk of onchocerciasis infection by human population movements between high and low transmission settings in Ghana

**DOI:** 10.1371/journal.pntd.0014039

**Published:** 2026-02-26

**Authors:** Sellase Pi-Bansa, Kwadwo Kyereme Frempong, Joseph Harold Nyarko Osei, Franklin Ayisi, Millicent Opoku, Millicent Selassie Afatodzie, Sampson Otoo, Sarah-Sally Mawunyo Dogbe, Abena Akyeamaa Nyarko, Aissatou Diawara, Sake de Vlas, Wilma Stolk, Daniel Adjei Boakye

**Affiliations:** 1 Department of Parasitology, Noguchi Memorial Institute for Medical Research, College of Health Sciences, University of Ghana, Legon, Ghana; 2 Biomedical and Public Health Research Unit, Water Research Institute, Council for Scientific and Industrial Research, Accra, Ghana; 3 African Regional Postgraduate Programme in Insect Science (ARPPIS), University of Ghana, Accra, Ghana; 4 Department of Ecological, Plant and Animal Sciences, School of Agriculture, Biomedicine and Environment, La Trobe University, Bundoora, Australia; 5 Global Institute for Disease Elimination, Abu Dhabi, United Arab Emirates; 6 Department of Public Health, Erasmus MC, University Medical Center Rotterdam, Rotterdam, Netherlands; 7 The END FUND, New York, New York, United States of America; George Washington University Medical Center, UNITED STATES OF AMERICA

## Abstract

**Background:**

Onchocerciasis control strategies have focused on mass drug administration (MDA) to reduce morbidity in high-risk (HR) areas (sites close to blackfly breeding sites). However, with the current drive towards elimination, treatment must be extended to low-risk (LR) areas. It is uncertain how well HR and LR communities are connected for decision making in recommending treatment strategies to include the LR areas. We provided data on current onchocerciasis infection status, connectivity between HR and LR communities and rates of human movement within some endemic communities in Ghana.

**Methods:**

Selected communities were 5km (HR) and 15km (LR) from breeding sites. Questionnaires were administered to obtain data on demographics and human movement patterns. Samples were collected from participants and tested for *O. volvulus* infections using Ov16 RDT and presence of microfilariae (mf) in skin snips using microscopy and quantitative PCR.

**Results:**

We observed a significantly higher onchocerciasis prevalence in HR than LR sites for both sero-prevalence (42.5% vrs 16.0%) and mf prevalence (15.2% vrs 4.1%) [*P* < 0.0001]. There was a high connectivity between the HR and LR with about 64–82% people who moved from HR to LR and 3.2-10% from LR to HR daily or weekly. Infection levels in those who moved from HR to LR communities were higher than those who moved from LR to HR, although these were not statistically significant (*P* > 0.05). Some individuals in Lancha (a LR community) who tested positive for infection frequently visited the HR communities for farming.

**Conclusions:**

A strong connectivity existed between HR and LR communities by human movement. On the average >60% of participants moved between endemic communities (between HR and LR) either daily, within the week or weekly. This supports the need to initiate treatment in LR areas; hence, such movement data would be useful during assessment of onchocerciasis elimination and delineation of transmission zones.

## Author summary

The main control of onchocerciasis is by MDA to reduce morbidity targeting only communities close to blackfly breeding sites where infection levels are high (high-risk, HR) sites. However, with a change from control to elimination, MDA should be extended to other sites within the endemic region which were not previously included, especially distant communities from breeding sites (low-risk, LR). It is not clear how people in the HR migrate to LR areas and vice versa. Such information will help formulate treatment strategies to include the LR sites towards the elimination agenda. To support such decisions, we collected human data on demographics and movement patterns in HR and LR endemic communities in Ghana. We also screened participants for onchocerciasis infection. Our findings show a higher onchocerciasis prevalence in HR than LR sites for both sero-prevalence (42.5% vrs 16.0%) and mf prevalence (15.2% vrs 4.1%) [P < 0.0001]. There was a strong connectivity between the HR and LR with about 64–82% who moved from HR to LR and 3.2-10% who moved from LR to HR daily or weekly. This highlights the need to equally treat LR areas; hence this movement information should be considered during assessment of onchocerciasis elimination and delineation of transmission zones.

## Introduction

Onchocerciasis is a neglected tropical disease (NTD) caused by the nematode *Onchocerca volvulus* and transmitted by blackflies [1]. Primary vectors of onchocerciasis in West/Central Africa and East Africa are members of the *Simulium* (*S.*) *damnosum* Theobald species complex and *S*. *neavei* respectively which breed in fast-flowing rivers [2,3]. In 2023 at least 249.5 million people required preventive treatment against onchocerciasis. As of 2017, approximately 14.6 million and 200,000 people worldwide had skin disease and confirmed completely blind, respectively, with Africa having the highest disease burden (99% of infected individuals) [4]. This mostly impacts affected individuals’ social and economic livelihoods, leading to poverty and eventually hindering their development [5].

The main strategy for onchocerciasis control is annual or biannual mass drug administration (MDA), targeting individuals aged 5 years and above who are not sick or pregnant [4]. The required MDA duration depends on different factors, including baseline endemicity, MDA frequency, MDA coverage and compliance patterns [6]. The initial aim of ivermectin MDA was to reduce morbidity due to onchocerciasis (control as a public health concern), and only onchocerciasis high-risk (HR) areas (hyper and meso-endemic; nodule prevalence ≥ 20%) were targeted for treatment [7], while low-risk (LR) areas (< 20% nodule prevalence) excluded. However, following reports of complete interruption of onchocerciasis transmission in endemic areas after years of MDA, the aim has shifted to elimination of transmission [8–10], hence the need to include LR areas in treatment.

Hypo-endemic or LR areas can be located at the boundaries of HR/meso-endemic sites from which infections are imported [11]. Human and blackfly movement could lead to importation of infection from HR to untreated LR areas which can spark resurgence of transmission. A recently published modelling study showed onchocerciasis elimination time depended on connectedness between villages either by human mobility or migrating flies [12]. To prevent such importation, MDA programs should be extended to LR areas, especially if these sites are spillover areas from neighbouring HR communities [12,13]. However, uncertainties about the level of connectedness and limited understanding of transmission dynamics in LR areas have hindered the development of treatment guidelines and criteria for starting MDA in these areas. This is of great concern to elimination programs since reinfection could occur in areas where transmission has been interrupted from neighbouring communities with ongoing infection.

This study was conducted to generate data to investigate the connectedness via mobile individuals between HR and LR communities. We have provided in this study the current infection status of onchocerciasis in selected endemic communities in Ghana, quantified the rates of human movement between HR and LR communities (to estimate connectedness), and determined some of the reasons associated with movement between these HR and LR communities.

## Materials and methods

### Ethics statement

Ethical approval of this study was sought from the institutional review board of the Noguchi Memorial Institute for Medical Research (Accra, Ghana; Study no. 055/21–22). All participants provided written consent to participate in the study.

### Study site

**Fig 1:**
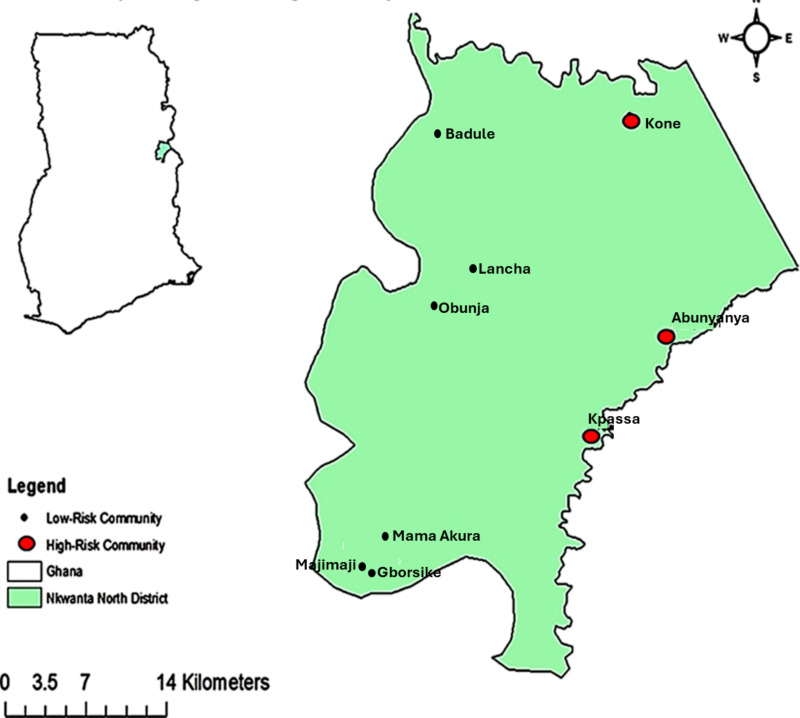
District map showing onchocerciasis low and high-risk communities selected within the Nkwanta North District. The base layer for this map was accessed from the “Simplemaps – Interactive Maps & Data” website [https://simplemaps.com/gis/country/gh]. License: Creative Commons Attribution 4.0. The software for drawing this map is ArcGIS v10.4.1 [www.esri.com]. The terms of use/ license information for the base layer image or shapefile is [https://data.humdata.org/about/license/legacy_hrinfo].

The study was conducted in nine communities located within the Nkwanta North District of the Oti Region, Ghana (Fig 1) from the year 2022–2023. The district lies between longitudes 00 10W and 00 45” E and latitudes 70 3” N and 80 45” N, with an estimated population of 126,096 as reported by the Ghana Statistical Service in 2021 [14]. The rainy season in this district spans from April to October while the dry season spans from November to March [15]. The mean annual rainfall ranges between 922mm and 1,874mm with relatively high mean humidity of 80%. The mean annual maximum and minimum temperatures range between 24°C to 39°C and 11°C to 26°C respectively [15]. Vegetation is predominantly characterized by Savannah woodland/grassland and occasional pockets of semi-deciduous forest [15]. Agriculture is the main economic activity in the district with about 80% of the population engaged in various aspects including crop farming, livestock, and fishing [15]. MDA commenced in majority of the communities within the district in the year 2000, and by the year 2017, the entire district had been covered by the mass treatment with ivermectin. A total of three communities were initially selected from HR communities (average microfilariae (mf) prevalence of 27%). Based on the HR communities, three LR communities (<5% mf prevalence) were also selected such that there was no physical barrier (e.g., mountain, water body, forest etc) between the HR and LR communities to inhibit the movement of blackflies and humans. Three additional LR communities were selected, making a total of six communities to make up for the relatively low participant numbers initially recorded. The selected HR and LR communities were 5 km (first line) and 15 km (third line) respectively from the blackfly breeding sites (Kpassa River Basin). The onchocerciasis HR communities selected for this study were Kone (8.70371’N latitude, 0.3391’E longitude), Abunyanya (8.55752’N latitude, 0.3702’E longitude) and Kpassa (Chief’s Palace/River View) (8.48794’N latitude, 0.30989’E longitude). The LR communities selected were Lancha (8.59564’N latitude, 0.21772’E longitude), Gborsike (8.4074’N latitude, 0.1519’E longitude) and Badule (8.69747’N latitude, 0.18981’E longitude). The additional communities were Majimaji (8.41077’N latitude, 0.14996’E longitude), Mama Akura (8.426’N latitude, 0.1592’E longitude) and Obunja (8.57597’N latitude, 0.17061’E longitude). All these nine communities had received 18 rounds of MDA before the commencement of the study.

### Study design

This was a cross-sectional survey. Eligible participants were individuals aged ≥ 20 years, residents in the communities, who provided written informed consent. Individuals < 20 years old, non-residents, invalids, and pregnant/lactating mothers were exempted. This study adopted the onchocerciasis elimination mapping protocol, which required screening of approximately 100 individuals or more from the study sites [16–18].

### Community engagement/Sensitization

This study leveraged on the existing Ghana Health Service (GHS) structure at the regional, district, and subdistrict levels, which already had an engagement with the various communities through the MDA activities by the NTD programme. All levels of the GHS hierarchy from national, through the regional to community were informed of the study. As part of the community entry, permission was sought from the chiefs and opinion leaders after a briefing on the study. Later, a durbar was organized involving the chiefs, elders, opinion leaders, health personnel from the various Community-Based Health Planning System (CHPS) compounds, community volunteers for ivermectin drug distribution, members of the community, etc. to educate them on the study and their role to enhance community participation. After the durbar, IEC (Information, Education, and Communication) in the form of announcements at the various community information centers and by the local information persons (“gong-gong” beater) were also utilized to inform the communities throughout the study.

### Questionnaire survey

A unique code was assigned to each participant after consenting to undergo the various procedures (questionnaire administration, blood sampling for dried blood spots (DBS), and skin snipping). A questionnaire was administered to the participants to collect their biodata (age, sex, occupation, marital status, educational background etc.), livelihood practices (doing house activities, going to work, attending social activities etc.) mobility patterns (their movement from LR to HR sites and vice versa, frequency of movement etc), ivermectin treatment history etc. Where necessary, community volunteers assisted with the interpretation of the questions into the local dialect of the participants.

### Blood sampling and processing for Dried Blood Spots (DBS)

About 60 µl of blood was sampled by finger pricking of the middle finger of each participant. Ten microliters of the blood was dropped on each of the 6 protrusions of a well-labelled Whatman filter disc. These were air-dried for at least 2 hours and packaged individually in an air-tight zip lock bag. All individual bags were packaged into a larger zip lock bag per community and stored at 4°C (using iced packs on the field then transported to the district health centre and stored in the laboratory fridge). Samples were later transferred to the cold chain at the Noguchi Memorial Institute for Medical Research (NMIMR) laboratory for further processing. OV-16 testing was later done by screening DBS of participants with SD Bioline onchocerciasis rapid test following manufacturers protocol [19,20].

### Screening of participants for infection using skin-snipping microscopy

Skin biopsy (skin snips) were collected from the two iliac crests around the waist of each participant [21,22]. The skin snips (SS) were then transferred into a 96-well microtiter plate containing 50 µl physiological saline (0.9%) and incubated at room temperature for 30 minutes before observing under a light microscope for the presence of mf. Samples were further confirmed 24 hours later. Individual SS (from both left and right iliac crest) were then transferred into 1.5ml tubes containing absolute ethanol for storage (-20°C) and further processing in the laboratory.

### Screening of skin snips using quantitative PCR

Skin snip samples preserved in absolute ethanol were centrifuged and the ethanol was decanted from the samples. DNA was extracted from the SS using the ZYMO DNA/RNA pathogen miniprep kit following the manufacturer’s protocol. DNA from samples was screened for the presence of *Onchocerca volvulus* targeting *Onchocerca volvulus-*specific 150 bp repeated DNA segments called O-150 using O-150 quantitative PCR on the Applied Biosystems 7300 Real-Time PCR system following the protocol of Mekonnen and colleagues [23].

### Collection of blackflies

Blackfly collections were done from 6 communities (3 HR and 3 LR communities). Community volunteers were recruited for fly collections. These volunteers included individuals who had participated in previous adult black fly collections. All the volunteers were trained in human landing catches technique, recording of data, storing and packaging of the blackfly samples. The collections were done for 6 months (from September 2022 to February 2023) using human landing catches [24]. The three first-line (closest to the breeding sites of vectors and with higher intensity of infections) communities; Abunyanya, Kone, and Kpassa which were 5 km from the breeding sites had 2 collection points (one at the breeding sites and another away from the breeding site, e.g., farm, bushes, etc.) with 2 collectors/site/day. The third line (farthest from breeding sites and with lower intensity of infections) communities; Badule, Lancha, and Gborsike which were 15 km or more from the breeding sites had 4 collection points (positioned in school park, farms, bushes, etc) also with 2 collectors/site/day. Collections were done once a week (four time in a month) in all study communities. The blackflies were preserved in absolute ethanol immediately after capturing. Vectors were collected and screened to confirm the presence, densities, and infection levels for a better understanding of the dynamics of transmission within the HR and LR communities.

### Screening of blackflies for O*nchocerca volvulus*

Adult flies were morphologically identified as forest or savannah following the protocol by Crosskey and colleagues [25]. The flies were then prepared in pools of up to 26 fly heads separated from pools of corresponding bodies according to communities, month of collection, and species (forest or savannah). Dissection of flies was done using entomological pins and a dissecting microscope. Heads were separated from the bodies of flies in a glass petri dish. The Petri dishes and the pins were washed thoroughly with 0.5% hypochlorite and later rinsed with 70% ethanol [26], before re-use. Pooled samples were placed in 2ml screw cap tubes and labelled accordingly. The heads and body pools were dried by incubating at 40°C for 30–60mins before DNA extraction. DNA was extracted using a Tissue/Insect Miniprep Kit following manufacturer’s protocol [26]. Pooled samples were analyzed using Real-time PCR to differentiate *O. volvulus* from *O. ochengi* [23] by amplification of an *Onchocerca* repeated sequence O-150*.* Reactions were carried out using Applied Biosystems 7500 v2.3 in a 15 µl reaction containing: the 0.4 µM of Primers (OVoo-NDS-FW and OVoo-NDS-Rev) and 0.2 µM of Probes, 2 µl of DNA template, 3.7 µl of nuclease free water and 1X Luna Universal probe qPCR Master Mix. Additionally, positive controls were used in every run (spiked fly pools with *O. volvulus* parasites), and pools of newly emerged flies from pupae collected from field used as negative control [23,27].

### Data analysis

Data were entered using Microsoft Excel (Microsoft Corporation; Redmond, WA, USA, version 2019) and imported into SPSS (version 29.0). The *O. volvulus* prevalence between the endemic communities (using microscopy, serology and qPCR) were determined using chi-square tests. The frequency of movement and reasons for the movement of participants were also analyzed using chi-square test. The movement was categorized based on responses from participants on where they travel daily, within the week, weekly, etc. Those communities were identified during data analyses as high-risk or low-risk. The risk of infection in individuals in relation to movement was based on the qPCR results. The poolscreen 2.0 software (University of Alabama; Birmingham, USA) was used to calculate the maximum likelihood estimate of infection in the vector population, along with the 95% confidence interval (CI). Poolscreen v2.0 [28] was used to estimate this maximum likelihood of *O. volvulus* infection rates in pools of unequal size. Outcomes were considered statistically significant at *P*-value ≤ 0.05.

## Results

### Questionnaire survey

A total of 752 participants were recruited, with 311 (41.4%) from HR and 441 (58.6%) from LR communities. Female participants were the majority with 434 (57.7%) and males were 318 (42.3%). Most of the study participants were married 584 (77.7%). In terms of education, about 62% had no formal education and with occupation, majority were farmers (73.6%), except in Gborsike which is a fishing community and Kpassa which is the district capital. Most participants (about 79%) had lived in the communities for over 10 years. The frequency of ivermectin intake by participants during onchocerciasis MDA was high, with >80% of participants indicating they had taken ivermectin at least once and about 41% taken more than 5 times prior to the study (Table 1).

**Table 1:** Demographics and characteristics of study participants.

Variable	High-risk communities			Low-risk communities						
Education	Abunyanya N (%)	Kpassa N (%)	Kone N (%)	Badule N (%)	Gborsike N (%)	Lancha N (%)	Majimaji N (%)	Mama Akura N (%)	Obunja N (%)	Overall N (%)
No formal Education	68 (61.8)	50 (48.1)	69 (71.1)	57 (68.7)	28 (48.3)	49 (57.0)	38 (63.3)	48 (66.7)	61 (74.4)	468 (62.2)
Pre-School	0 (0.0)	1 (1.0)	0 (0.0)	1 (1.2)	0 (0.0)	0 (0.0)	0 (0.0)	0 (0.0)	0 (0.0)	2 (0.3)
Primary	29 (26.4)	41 (39.4)	20 (20.6)	14 (16.9)	21 (35.2)	23 (26.7)	17 (28.3)	18 (25.0)	16 (19.5)	199 (26.5)
Secondary	12 (10.9)	10 (9.6)	6 (6.2)	9 (10.8)	8 (13.8)	13 (15.1)	5 (8.3)	4 (5.6)	2 (2.4)	69 (9.2)
Tertiary	1 (0.9)	2 (1.9)	2 (2.1)	2 (2.4)	1 (1.7)	1 (1.2)	0 (0.0)	2 (2.8)	3 (3.7)	14 (1.9)
**Occupation**										
Farmer	92 (83.6)	50 (48.1)	83 (85.6)	62 (74.7)	8 (13.8)	70 (81.4)	53 (88.3)	64 (88.9)	73 (89.0)	554 (73.8)
Student	4 (3.6)	2 (1.9)	2 (2.1)	7 (8.4)	3 (5.2)	7 (8.1)	2 (3.3)	2 (2.8)	4 (4.9)	33 (4.4)
Fisherman/Fishmonger	0 (0.0)	0 (0.0)	0 (0.0)	0 (0.0)	19 (32.8)	0 (0.0)	0 (0.0)	1 (1.4)	0 (0.0)	20 (2.7)
Teacher	0 (0.0)	2 (1.9)	0 (0.0)	1 (1.2)	0 (0.0)	0 (0.0)	0 (0.0)	0 (0.0)	0 (0.0)	3 (0.4)
Trader	4 (3.6)	9 (8.7)	5 (5.2)	1 (1.2)	16 (27.6)	1 (1.2)	0 (0.0)	0 (0.0)	0 (0.0)	36 (4.8)
Unemployed	3 (2.7)	9 (8.7)	2 (2.1)	3 (3.6)	1 (1.7)	0 (0.0)	2 (3.3)	1 (1.4)	1 (1.2)	22 (2.9)
Health worker	0 (0.0)	0 (0.0)	1 (1.0)	1 (1.2)	0 (0.0)	0 (0.0)	0 (0.0)	0 (0.0)	1 (1.2)	3 (0.4)
Other	7 (6.4)	32 (30.8)	4 (4.1)	8 (9.6)	11 (19.0)	8 (9.3)	3 (5.0)	4 (5.6)	3 (3.7)	80 (10.6)
**Duration lived in Community**										
< 1 year	2 (1.8)	2 (1.9)	0 (0.0)	0 (0.0)	7 (12.1)	2 (2.3)	1 (1.7)	1 (1.4)	0 (0.0)	15 (2.0)
1–5 years	1 (0.9)	13 (12.5)	7 (7.2)	5 (6.0)	11 (19.0)	10 (11.6)	12 (20.0)	11 (15.3)	8 (9.8)	78 (10.4)
6–10 years	2 (1.8)	15 (14.4)	5 (5.2)	5 (6.0)	6 (10.3)	11 (12.8)	7 (11.7)	8 (11.1)	6 (7.3)	65 (8.6)
11–17 years	42 (38.2)	57 (54.8)	52 (53.6)	31 (37.3)	25 (43.1)	31 (36.5)	29 (48.3)	27 (37.5)	37 (45.1)	331 (44.0)
From birth (>=18 years)	63 (57.3)	17 (16.3)	33 (34.0)	42 (50.6)	9 (15.5)	32 (37.2)	11 (18.3)	25 (34.7)	31 (37.8)	263 (35)
**Frequency of IVM intake**										
None	6 (5.5)	73 (72.3)	3 (3.1)	6 (7.2)	10 (17.2)	1 (1.2)	1 (1.7)	1 (1.4)	1 (1.2)	102 (13.6)
1–5 times	93 (84.5)	27 (26.7)	23 (23.7)	50 (60.2)	16 (27.6)	44 (51.2)	41 (68.3)	36 (50)	10 (12.2)	340 (45.4)
6–10 times	11 (10)	0 (0.0)	54 (55.7)	24 (28.9)	20 (34.5)	29 (33.7)	15 (25.0)	28 (38.9)	43 (52.4)	224 (29.9)
> 10 times	0 (0.0)	1 (1.0)	17 (17.5)	3 (3.6)	12 (20.7)	12 (14.0)	3 (5.0)	7 (9.7)	28 (34.1)	83 (11.1)
**Marital Status**										
Cohabitation	0 (0)	0 (0)	0 (0)	1 (1.2)	1 (1.8)	0 (0)	0 (0)	0 (0)	0 (0)	2 (0.3)
Divorced	0 (0)	1 (1)	2 (2.1)	0 (0)	0 (0)	0 (0)	0 (0)	0 (0)	0 (0)	3 (0.4)
Married	85 (77.3)	78 (75.0)	76 (79.2)	53 (63.9)	46 (80.7)	67 (77.9)	47 (78.3)	65 (90.3)	67 (81.7)	584 (77.9)
Separated	1 (0.9)	1 (1.0)	0 (0)	0 (0)	0 (0)	0 (0)	0 (0)	0 (0)	0 (0)	2 (0.3)
Single	20 (18.2)	14 (13.5)	18 (18.8)	23 (27.7)	9 (15.8)	18 (20.9)	9 (15.0)	4 (5.6)	10 (12.2)	126 (16.8)
Widowed	4 (3.6)	10 (9.6)	0 (0.0)	6 (7.2)	1 (1.7)	1 (1.2)	4 (6.7)	3 (4.2)	5 (6.1)	34 (4.5)

N = number of respondents to a particular question. Figures in parenthesis represent percentages of the respondents out of the total number of participants in each community. Total number of participants from study communities was 752. These are comprised of sampling from the various communities as follows: Abunyanya = 110, Kpassa = 104, Kone = 97, Badule = 83, Gborsike = 58, Lancha = 86, Majimaji = 60, Mama Akura = 72, Obunja = 82

### Infection prevalence in humans and vectors in the study sites

#### Infection prevalence in humans.

A total of 752 participants were examined with 311 from HR and 441 from LR communities. There was a significantly higher sero-prevalence in participants from HR than LR, and in males than females [HR was 42.5% (127/299) (95% CI = 37% – 48%) compared to LR which was 16.0% (67/420) (95% CI = 12% – 19%) with *P* = 0.0001. Prevalence in males was 35.4% (108/305) (95% CI = 19.33% – 63.03%) and females 20.8% (86/414) (95% CI = 26.46% – 66.01%) with *P* = 0.0001.

Skin snipped samples screened for *O. volvulus* parasites by microscopy showed 5.9% (18/306) (95% CI = 3% – 9%) and 0.9% (4/429) (95% CI = 0% – 2%) mf prevalence for HR and LR communities, respectively. This indicates a significantly higher mf prevalence for HR than LR communities (*P* = 0.0001)**.** Additionally, the mf prevalence in males and females was 4.5% (14/309) (95% CI = 2% – 7%) and 1.9% (8/426) (95% CI = 1% – 3%) (*P* = 0.037), respectively. The skin snipped samples were further screened with O-150 qPCR. The results indicated more positives with 15.2% (42/276) (95% CI = 11% – 19%) prevalence in HR and 4.1% (14/344) (95% CI = 2% – 6%) in LR communities. This shows a significant difference in mf prevalence (*P* = 0.0001) for the study sites. The mf prevalence in males was higher at 11.7% (31/264) (95% CI = 8% – 16%) than in females at 7.0% (25/365) (95% CI = 4% – 10%) following qPCR (*P* = 0.043)**.** The community specific prevalences have been summarized in Table 2.

**Table 2:** Infection prevalence in humans and vectors from study communities.

			Prevalence in humans			Blackfly infectivity					
Endemicity level	Community	No. examined	Ov16 Serology (%)	Skin snip Microscopy (%)	Skin snip qPCR (%)	No. of blackflies	No. of pools examined	Head: No. of positive pools	Head: MLE (95% CI)	Body: No. of positive pools	Body: MLE (95% CI)
**HR**	Abunyanya	110	49.5 ^a^	6.5 ^a^	19.4 ^a^	202	13	1	0.0053 (0.0002 – 0.0268)	6	0.0452 (0.0152 – 0.1019)
	Kpassa	104	26.5 ^b^	5.9 ^a^	16.0 ^a^	827	44	4	0.0051 (0.0013 – 0.0131)	10	0.0137 (0.0061 – 0.0257)
	Kone	97	52.2 ^a^	5.2 ^a^	8.9 ^b^	515	27	1	0.0020 (0.0006 – 0.0101)	4	0.0084 (0.0021 – 0.0216)
**LR**	Badule	83	16 ^b^	0.0 ^b^	7.2 ^b^	16	6	0	0.0000 (0.0000 – 0.1130)	0	0.0000 (0.0000 – 0.1130)
	Gborsike	58	9.8 ^b^	0.0 ^b^	6.7 ^a^	6	2	0	0.0000 (0.0000 – 0.1130)	0	0.0000 (0.0000 – 0.1130)
	Lancha	86	13.9 ^b^	4.7 ^a^	5.8 ^b^	20	6	0	0.0000 (0.0000 – 0.1130)	0	0.0000 (0.0000 – 0.1130)
	Majimaji	60	25.8 ^b^	0.0 ^b^	0.0 ^b^	NA	NA	NA	NA	NA	NA
	Mama Akura	72	14.3 ^b^	0.0 ^b^	5.1 ^b^	NA	NA	NA	NA	NA	NA
	Obunja	82	16.3 ^b^	0.0 ^b^	0.0 ^b^	NA	NA	NA	NA	NA	NA

NB: Abunyanya was used as the reference community for comparison with other communities since this community has been one of the communities with recent infections based on data from the NTD programme, Ghana and recorded highest prevalence in this study. a: indicates prevalence not statistically different (*P* > 0.05) from that of the reference community (Abunyanya) b: indicates prevalence statistically different (*P* ≤ 0.05) from that of the reference community (Abunyanya). HR = High-risk community; LR = Low-risk community. MLE = Maximum Likelihood Estimate (gives an estimation of the infection rate per fly); CI = Confidence interval. NA = Not Applicable; No fly collection point was set up in the community

#### Infection prevalence in blackflies.

A total of 1,568 adult blackflies were collected from the 3 HR and 3 LR communities. The highest number of flies were captured in Kpassa 737/1,568 (47.0%), followed by 581/1,568 (37.1%) and 212/1,568 (13.5%) from Kone and Abunyanya, respectively. For these communities, the highest numbers were caught in January 2023. The lowest numbers were collected in Gborsike 6/1,568 (0.4%). Blackflies were identified as forest 774/1,568 (49.4%) and savannah 794/1,568 (50.6%) species

Additionally, pool screening of the flies for filarial worm detection recorded at least one positive pool for head and body pools for HR communities. Kpassa recorded the highest number of positive pools with *O. volvulus* infection detected in 4/44 (95% CI = 0.13% – 1.32%) pooled blackfly heads and 8/44 (95% CI = 0.52% – 2.36%) pools of blackfly bodies. A positive pool of bodies was interpreted as infected with mf or developing *O. volvulus* larvae, whereas a positive pooled heads was inferred as containing infective L3 (larval stage 3) parasites. No positive pool was recorded for pooled blackfly heads and bodies for all the LR communities. Details of blackfly infectivity levels with *O. volvulus* within the Low and High-risk communities are found in the S1 and S2 Tables.

### Movement patterns of participants between communities

**Fig 2:**
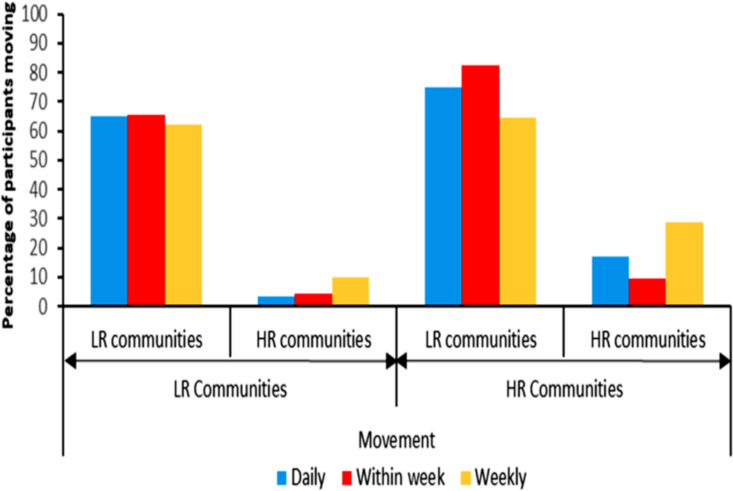
Movement of participants from HR to LR and LR to HR communities daily, within the week, and weekly. The LR communities or HR communities below the horizontal arrows indicate the sites where participants moved from. The LR communities or HR communities above the horizontal arrows (under the bars) indicate the sites where they moved to.

**Fig 3:**
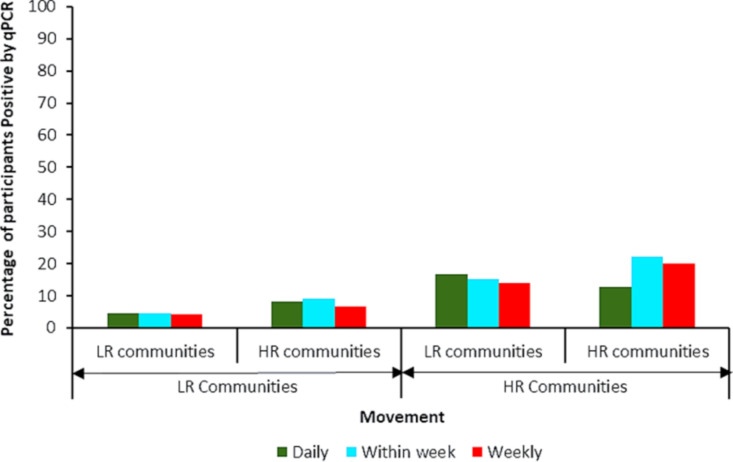
*Onchocerca volvulus* infection in participants based on movements. The LR communities or HR communities below the horizontal arrows indicate the sites where participants moved from. The LR communities or HR communities above the horizontal arrows (under the bars) indicate the sites where they moved to.

A higher percentage of participants (74.9%) indicated they moved from HR to LR communities daily. Those who indicated they moved some days within the week were 82.3% and those who indicated they moved once a week (weekly) were 64.6% (N = 311) [Fig 2]. On the other hand, those who moved from LR to HR communities daily were 3.2%, some days within the week were 4.3% and once a week were 10% (N = 441) [Fig 2]. We assessed infection levels among those who moved across the different endemicity settings (HR & LR). The infection levels (by qPCR) in the group of individuals who indicated they moved from HR to LR communities were higher for [daily 16.7% (35/209), within the week 15.3% (35/229), weekly 14.0% (25/178)] compared to those who moved from LR to HR [daily 8.3% (1/12), within the week 9.1% (1/11), weekly 6.7% (2/30)]. Nonetheless, these were not statistically significant [daily (Pearson chi-square = 0.589; df = 1; *P* = 0.443), within the week (Pearson chi-square = 0.316; df = 1; *P* = 0.574), weekly ((Pearson chi-square = 1.237; df = 1; *P* = 0.266)] [Fig 3]. The study participants provided various reasons for their movements to other communities which included visiting family and friends, trading, farming, recreational activities, to the market etc.

## Discussion

Elimination of onchocerciasis as a public health problem requires extending treatment to all endemic areas apart from the high endemic sites (close to fly breeding locations). The initial strategy which excluded other endemic areas such as low-risk (LR) sites (≥ 15 km from fly breeding locations) could be problematic since this can lead to resurgence of the disease in areas previously controlled [29]. It is important to highlight that as the NTD programmes change policy from control to elimination of onchocerciasis, there is the need to reexamine the contribution of LR or hypoendemic areas in sustaining infections by migration [11,30–32]. This study was conducted in the Nkwanta North District, Ghana with study sites selected from onchocerciasis LR and HR areas based on data from the NTD programme. The results from this study indicated that onchocerciasis prevalence in participants from the HR communities was higher than those from the LR. Also, a higher prevalence was observed screening samples with qPCR due to the high sensitivity after examination by microscopy. This result is consistent with previous studies that also reported similar findings [32–35].

In general, male participants presented with relatively higher *O. volvulus* infections than the females. The high prevalence observed in males could be attributed to their occupational lifestyle which predisposed them to infections. The male participants during the survey indicated they were mostly involved in farming or fishing, which mostly kept them outdoors, exposing them to infective blackfly bites. Previous studies have also reported males to be more predisposed to blackfly bites increasing their risk of infections [36–40]. The females in our study indicated they were primarily engaged in trading at the market or housekeeping, hence, less contact with the vectors and reduction in risk of infection.

We also showed a high level of connectivity between HR and LR communities by movement of inhabitants daily and weekly. The participants observed moving from the HR to LR sites daily, within the week, and weekly were higher compared to movement from LR to HR sites. This may be mainly related to trading activities, since majority were farmers and fishermen in the HR sites and had to move their goods from the farm for trading frequently outside the vicinity to other locations which are mainly distant from the fly breeding sites (LR sites). On the other hand, the few participants residing in LR and visiting HR sites were mainly because they had their farms there. In general, a greater migration by study participants was to the LR communities because these areas tend to be more urbanised with much more commercial activities, and also close to the Oti water body where fish trading and transportation are vibrant (Apart from Kpassa, the other HR communities were more rural).

We also observed that a higher percentage of participants from HR to LR sites daily, within the week, and weekly tested positive for *O. volvulus* infection compared to those who moved from LR to HR. This is expected since those living in the HR sites get a lot more bites from the flies and hence higher infections in this group. Nonetheless, since the blackfly densities in the LR sites are low, and these farmers mostly move for trading activities (short lived), their risk of spreading the disease in the LR sites is low (Table 2). The concerns are those living in the LR sites where there is no treatment and move to the HR sites to pick infections and sustain transmission. Our data suggest that the few people moving from LR to HR sites spent ample time in the HR communities (mainly for farming and fishing utilizing the rivers) where they are exposed to blackfly bites and pick up infections (Figs 2 and 3). We showed in our results that four participants from Lancha (LR community) were positive for *O. volvulus* parasite. These positive participants were all farmers and indicated they frequently moved to HR communities daily and weekly, spending between 2–6 hours in these communities. Although various activities require movement between LR and HR areas, those related to farming were very important because they were mostly daily and of longer duration. Hence, if farms are within 5–10 km from the breeding sites, the farmers will be exposed to high blackfly biting intensities and infections. Stapley and colleagues who modelled transmission thresholds and hypoendemic stability for onchocerciasis elimination reported similar observation [41].

We showed that 72% of the respondents from Kpassa, a HR community indicated they had never received treatment. This may be due to the fact that Kpassa is the capital of the Nkwanta North District and an urbanized area with relatively large population size. MDA activities in Kpassa are mostly limited to areas close to the breeding sites (Kpassa river), leaving many people who live further away not treated. Additionally, most individuals are absent during treatment due to other engagements and travels thereby missing treatment.

The densities of flies are important in sustaining transmission, and these are associated with the proximity to breeding sites [39–42]. We observed higher numbers of *Simulium damnosum* recorded from the HR communities compared to LR as represented in Table 2. This observation of high fly numbers in the HR sites was associated with the proximity of these communities to the Kpassa river (which is a breeding source for blackflies) since the HR sites were selected 5 km away from the river compared to 15 km or more away for the LR sites. Ayisi et al. investigating onchocerciasis transmission at the Cameroon-Chad border area after more than 20 years of annual MDA observed that continual transmission of human onchocerciasis in some study sites were due to their proximity to river bodies [42]. These proximity of fly breeding sites to human supports the high infections in the fly pools we examined from the HR with no infections in flies from the LR communities. Also, for the positivity of blackfly pools in all HR communities, we observed higher infection in the body of flies, which as expected may be due to the presence of different stages, i.e., L1, L2, or L3 compared to infectivity in the head which mostly harbour only the L3 stage of parasite. Similar findings have also been reported by other studies [26,43]. It should be noted that the reporting of infective fly heads in HR communities is indicative of active ongoing onchocerciasis transmission in these areas. The abundance of vectors with high vector capacity, efficiency and the availability of infected individuals lead to continuous and sustained disease transmission [30,44,45]. We have shown that the journey towards elimination of onchocerciasis must factor in treating all communities within the endemic district including areas classified as LR or hypoendemic, since movement of humans between the HR and LR sites could sustain infections and prolong the elimination timelines.

## Conclusion

Our data showed a high level of connectivity between HR and LR communities by human movement. On the average >60% of participants moved between endemic communities (between HR and LR) either daily, within the week or weekly. Our findings indicated ongoing transmission especially within the HR communities. Infected individuals in LR communities may not necessarily pose a transmission risk in their area due to absence or low vector density, but, due to their movements, may serve as reservoir for re-introduction of infection in the HR areas. Occupations such as farming and fishing close to blackfly breeding sites poses higher risk to residents who moved to work in these sites. MDA need to be extended to LR sites especially where they are connected by human migration. Therefore, data on human movements should be considered during assessment of onchocerciasis elimination in endemic communities and the delineation of transmission zones.

## Supporting information

S1 Table Blackfly infectivity levels with *O. volvulus* parasites within the Low-risk communities. (DOCX)

S2 Table Blackfly infectivity levels with *O. volvulus* parasites within the High-Risk Communities. (DOCX)
