# Supplementary material for: The risk of onchocerciasis infection by human population movements between high and low transmission settings in Ghana
Source: PLoS Negl Trop Dis. 2026 Feb 26;20(2):e0014039. doi: 10.1371/journal.pntd.0014039 (PMC12962500; doi:10.1371/journal.pntd.0014039)
Supplement: S1 Table — (DOCX) [file pntd.0014039.s001.docx]

**S1 Table. Blackfly infectivity levels with *O. volvulus* parasites within the Low-Risk Communities**

| **Lancha** | | | | | **Badule** | | | | | **Gborsike** | | | | |
| --- | --- | --- | --- | --- | --- | --- | --- | --- | --- | --- | --- | --- | --- | --- |
|  |  |  | **Infectivity** | **Infection** |  |  |  | **Infectivity** | **Infection** |  |  |  | **Infectivity** | **Infection** |
| **Month of**  **collection** | **Pooled species** | **Flies per pool** | **+VE/-VE Pool**  **(Head)** | **+VE/-VE Pool**  **(Body)** | **Month of collection** | **Pooled species** | **Flies per pool** | **+VE/-VE Pool**  **(Head)** | **+VE/-VE Pool**  **(Body)** | **Month of collection** | **Pooled species** | **Flies per pool** | **+VE/-VE Pool**  **(Head)** | **+VE/-VE Pool**  **(Body)** |
| Dec, 2022 | Savannah | 1 | Negative | Negative | Jan, 2023 | Forest | 2 | Negative | Negative | Sept,2022 | Forest | 2 | Negative | Negative |
| Nov, 2022 | Forest | 1 | Negative | Negative | Jan, 2023 | Savannah | 1 | Negative | Negative | Sept,2022 | Savannah | 4 | Negative | Negative |
| Oct, 2022 | Forest | 4 | Negative | Negative | Dec, 2022 | Savannah | 5 | Negative | Negative |  |  |  |  |  |
| Oct, 2022 | Savannah | 4 | Negative | Negative | Dec, 2022 | Forest | 1 | Negative | Negative |  |  |  |  |  |
| Sept,2022 | Forest | 7 | Negative | Negative | Nov,2022 | Savannah | 4 | Negative | Negative |  |  |  |  |  |
| Sept,2022 | Savannah | 3 | Negative | Negative | Sept,2022 | Forest | 3 | Negative | Negative |  |  |  |  |  |
|  |  |  |  |  |  |  |  |  |  |  |  |  |  |  |
| **Total** | | **20** |  |  | **Total** | | **16** |  |  | **Total** | | **6** |  |  |
