# Supplementary material for: The risk of onchocerciasis infection by human population movements between high and low transmission settings in Ghana
Source: PLoS Negl Trop Dis. 2026 Feb 26;20(2):e0014039. doi: 10.1371/journal.pntd.0014039 (PMC12962500; doi:10.1371/journal.pntd.0014039)
Supplement: S2 Table — (DOCX) [file pntd.0014039.s002.docx]

**S2 Table. Blackfly infectivity levels with *O. volvulus* parasites within the High-Risk Communities**

| **Abunyanya** | | | | | **Kpassa** | | | | | **Kone** | | | | |
| --- | --- | --- | --- | --- | --- | --- | --- | --- | --- | --- | --- | --- | --- | --- |
|  |  |  | **Infectivity** | **Infection** |  |  |  | **Infectivity** | **Infection** |  |  |  | **Infectivity** | **Infection** |
| **Month of**  **collection** | **Pooled species** | **Flies per pool** | **+VE/-VE Pool**  **(Head)** | **+VE/-VE Pool**  **(Body)** | **Month of collection** | **Pooled species** | **Flies per pool** | **+VE/-VE Pool**  **(Head)** | **+VE/-VE Pool**  **(Body)** | **Month of collection** | **Pooled species** | **Flies per pool** | **+VE/-VE Pool**  **(Head)** | **+VE/-VE Pool**  **(Body)** |
| Feb, 2023 | Forest | 20 | Negative | Positive | Feb, 2023 | Forest | 20 | Negative | Negative | Feb, 2023 | forest | 20 | Negative | Negative |
| Feb, 2023 | Forest | 11 | Negative | Positive | Feb, 2023 | Forest | 20 | Negative | Negative | Feb, 2023 | forest | 21 | Negative | Negative |
| Feb, 2023 | Savannah | 24 | Positive | Positive | Feb, 2023 | Forest | 20 | Negative | Negative | Feb, 2023 | forest | 20 | Negative | Negative |
| Jan, 2023 | Forest | 24 | Negative | Positive | Feb, 2023 | Forest | 20 | Negative | Negative | Feb, 2023 | forest | 13 | Negative | Negative |
| Jan, 2023 | Forest | 25 | Negative | Positive | Feb, 2023 | Forest | 20 | Negative | Negative | Jan, 2023 | forest | 20 | Negative | Negative |
| Jan, 2023 | Savannah | 20 | Negative | Positive | Feb, 2023 | Savannah | 20 | Negative | Negative | Jan, 2023 | forest | 20 | Negative | Negative |
| Jan, 2023 | Savannah | 19 | Negative | Negative | Feb, 2023 | Savannah | 20 | Negative | Positive | Jan, 2023 | forest | 20 | Negative | Positive |
| Dec, 2022 | Forest | 25 | Negative | Negative | Feb, 2023 | Savannah | 20 | Negative | Negative | Jan, 2023 | forest | 20 | Negative | Negative |
| Dec, 2022 | Savannah | 17 | Negative | Negative | Feb, 2023 | Savannah | 11 | Negative | Negative | Jan, 2023 | forest | 25 | Negative | Negative |
| Nov, 2022 | Forest | 2 | Negative | Negative | Jan, 2023 | Forest | 20 | Negative | Negative | Jan, 2023 | savannah | 20 | Negative | Negative |
| Nov, 2022 | Savannah | 5 | Negative | Negative | Jan, 2023 | Forest | 20 | Negative | Negative | Jan, 2023 | savannah | 20 | Negative | Negative |
| Oct, 2022 | Forest | 6 | Negative | Negative | Jan, 2023 | Forest | 20 | Negative | Positive | Jan, 2023 | savannah | 20 | Negative | Negative |
| Oct, 2022 | Savannah | 4 | Negative | Negative | Jan, 2023 | Forest | 20 | Negative | Positive | Dec, 2022 | forest | 22 | Negative | Negative |
|  |  |  |  |  | Jan, 2023 | Forest | 20 | Positive | Negative | Dec, 2022 | forest | 24 | Negative | Negative |
|  |  |  |  |  | Jan, 2023 | Forest | 20 | Negative | Negative | Dec, 2022 | savannah | 23 | Negative | Negative |
|  |  |  |  |  | Jan, 2023 | Forest | 13 | Negative | Positive | Dec, 2022 | savannah | 24 | Negative | Negative |
|  |  |  |  |  | Jan, 2023 | Forest | 24 | Negative | Negative | Nov,2022 | forest | 3 | Negative | Negative |
|  |  |  |  |  | Jan, 2023 | Forest | 21 | Negative | Negative | Nov,2022 | savannah | 20 | Positive | Negative |
|  |  |  |  |  | Jan, 2023 | Forest | 22 | Negative | Negative | Nov,2022 | savannah | 7 | Negative | Negative |
|  |  |  |  |  | Jan, 2023 | Forest | 13 | Negative | Negative | Oct, 2022 | forest | 3 | Negative | Negative |
|  |  |  |  |  | Jan, 2023 | Savannah | 20 | Negative | Negative | Oct, 2022 | savannah | 20 | Negative | Negative |
|  |  |  |  |  | Jan, 2023 | Savannah | 20 | Negative | Negative | Oct, 2022 | savannah | 21 | Negative | Negative |
|  |  |  |  |  | Jan, 2023 | Savannah | 20 | Negative | Negative | Sept,2022 | forest | 25 | Negative | Negative |
|  |  |  |  |  | Jan, 2023 | Savannah | 20 | Negative | Negative | Sept,2022 | forest | 26 | Negative | Positive |
|  |  |  |  |  | Jan, 2023 | Savannah | 20 | Negative | Negative | Sept,2022 | savannah | 20 | Negative | Negative |
|  |  |  |  |  | Jan, 2023 | Savannah | 20 | Negative | Negative | Sept,2022 | savannah | 20 | Negative | Negative |
|  |  |  |  |  | Jan, 2023 | Savannah | 20 | Positive | Positive | Sept,2022 | savannah | 18 | Negative | Positive |
|  |  |  |  |  | Jan, 2023 | Savannah | 20 | Positive | Positive |  |  |  |  |  |
|  |  |  |  |  | Jan, 2023 | Savannah | 12 | Negative | Negative |  |  |  |  |  |
|  |  |  |  |  | Jan, 2023 | Savannah | 20 | Negative | Negative |  |  |  |  |  |
|  |  |  |  |  | Jan, 2023 | Savannah | 13 | Negative | Negative |  |  |  |  |  |
|  |  |  |  |  | Dec, 2022 | Forest | 20 | Negative | Negative |  |  |  |  |  |
|  |  |  |  |  | Dec, 2022 | Forest | 20 | Negative | Negative |  |  |  |  |  |
|  |  |  |  |  | Dec, 2022 | Forest | 20 | Negative | Positive |  |  |  |  |  |
|  |  |  |  |  | Dec, 2022 | Savannah | 20 | Negative | Negative |  |  |  |  |  |
|  |  |  |  |  | Dec, 2022 | Savannah | 20 | Negative | Negative |  |  |  |  |  |
|  |  |  |  |  | Dec, 2022 | Savannah | 25 | Positive | Negative |  |  |  |  |  |
|  |  |  |  |  | Nov,2022 | Forest | 20 | Negative | Negative |  |  |  |  |  |
|  |  |  |  |  | Nov,2022 | Forest | 24 | Negative | Negative |  |  |  |  |  |
|  |  |  |  |  | Nov,2022 | Savannah | 12 | Negative | Negative |  |  |  |  |  |
|  |  |  |  |  | Nov,2022 | Savannah | 20 | Negative | Positive |  |  |  |  |  |
|  |  |  |  |  | Oct, 2022 | Forest | 10 | Negative | Negative |  |  |  |  |  |
|  |  |  |  |  | Oct, 2022 | Savannah | 20 | Negative | Negative |  |  |  |  |  |
|  |  |  |  |  | Oct, 2022 | Savannah | 7 | Negative | Negative |  |  |  |  |  |
|  |  |  |  |  |  |  |  |  |  |  |  |  |  |  |
| **Total** | | **202** |  |  | **Total** | | **827** |  |  | **Total** | | **515** |  |  |

A supplementary table showing blackfly pool composition, species numbers, months of collection and fly numbers
